# Supplementary figures and images for: Microglia and macrophages alterations in the CNS during acute SIV infection: A single-cell analysis in rhesus macaques
Source: PLoS Pathog. 2024 Sep 16;20(9):e1012168. doi: 10.1371/journal.ppat.1012168 (PMC11426456; doi:10.1371/journal.ppat.1012168)

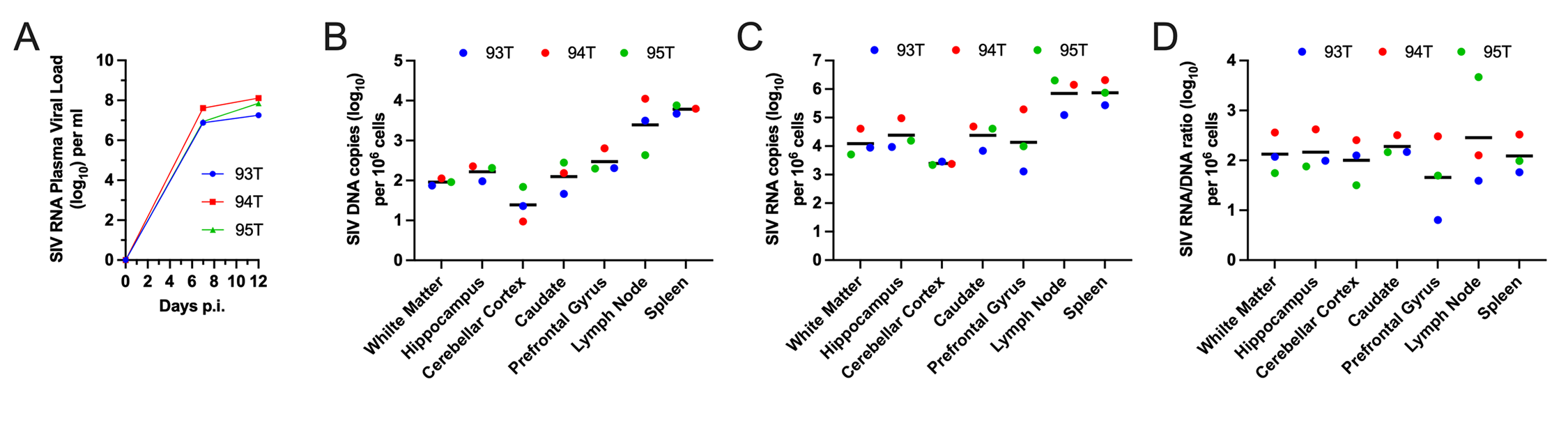

Supplement: S1 Fig — (TIF) [file ppat.1012168.s001.tif]

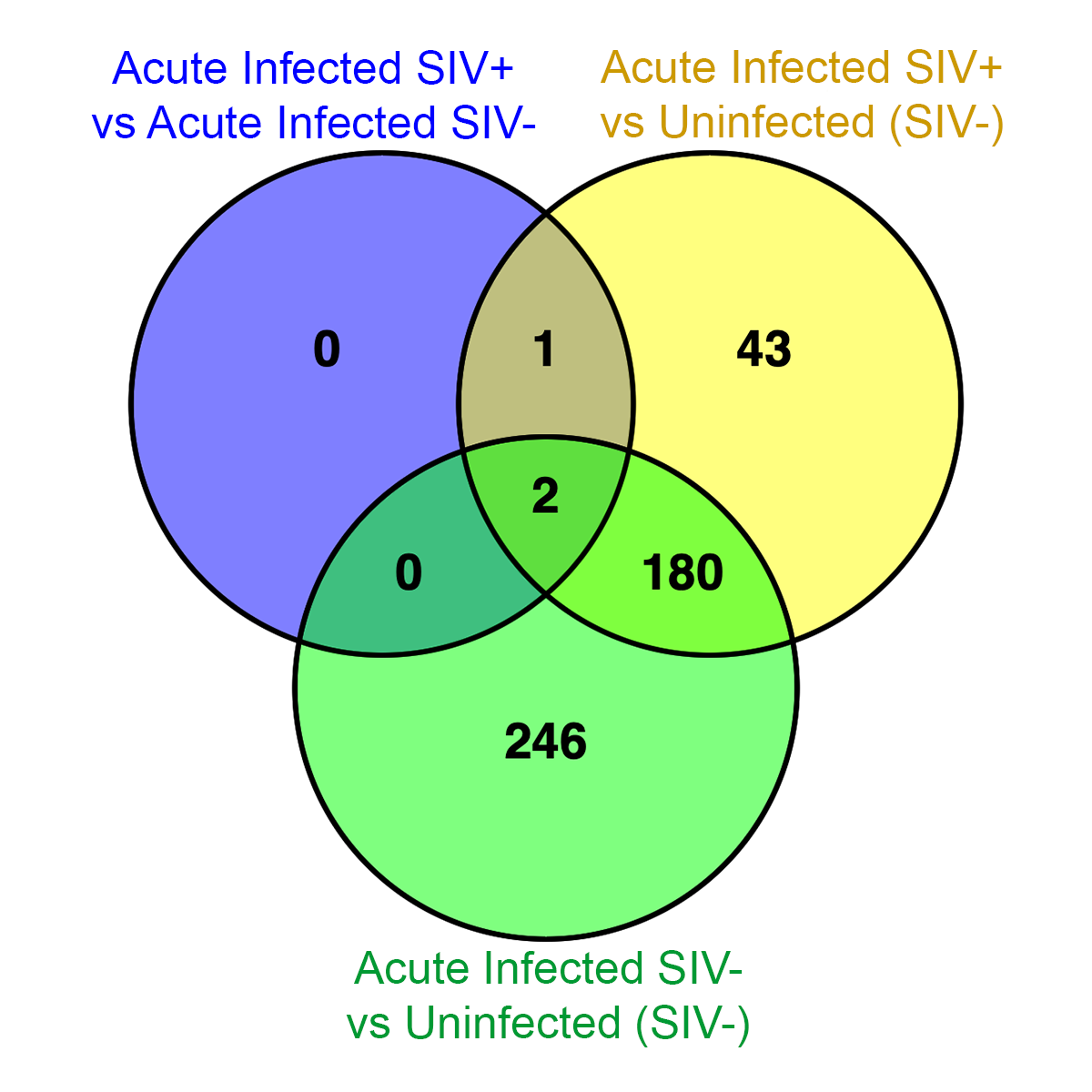

Supplement: S2 Fig — (TIF) [file ppat.1012168.s002.tif]

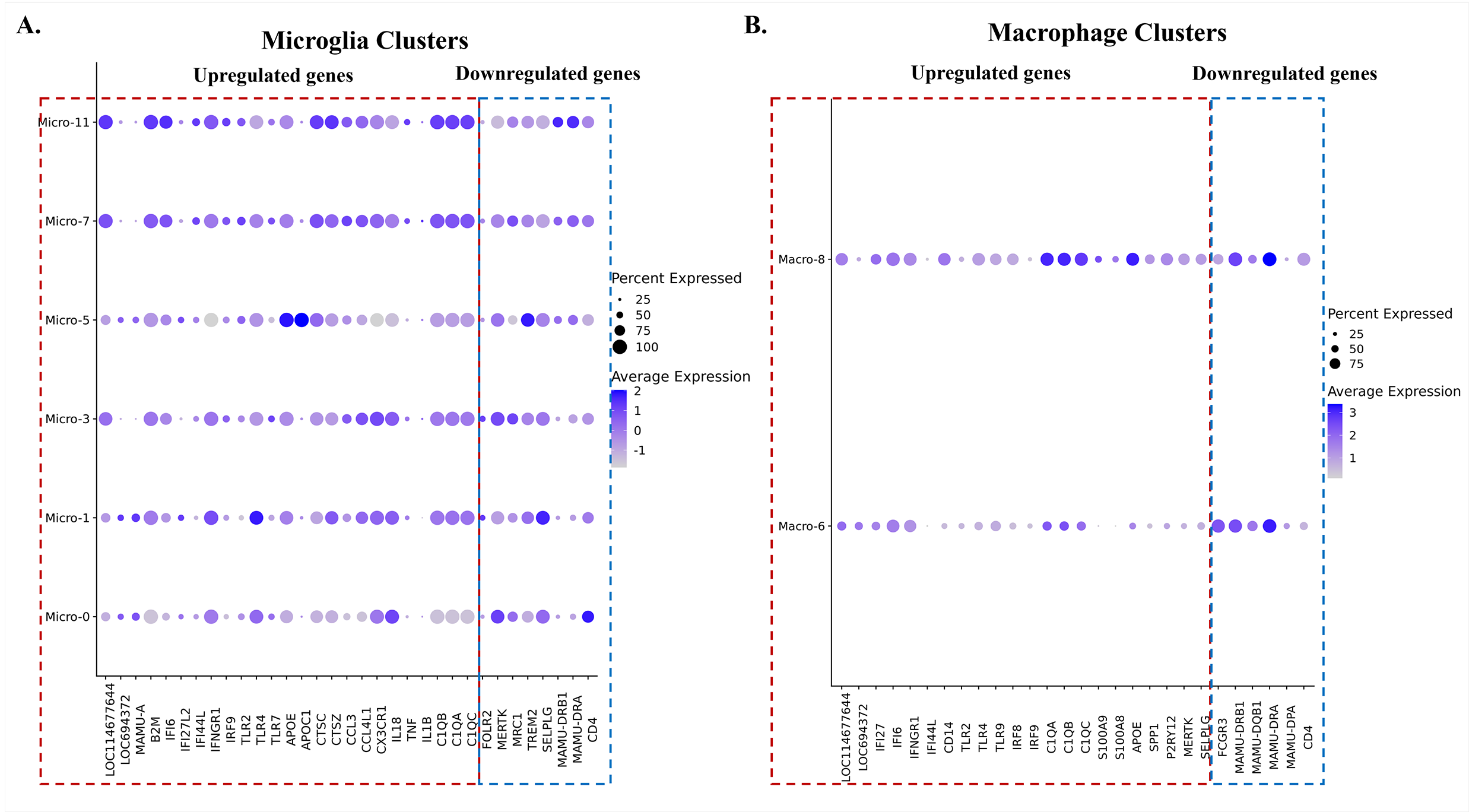

Supplement: S3 Fig — (TIF) [file ppat.1012168.s003.tif]

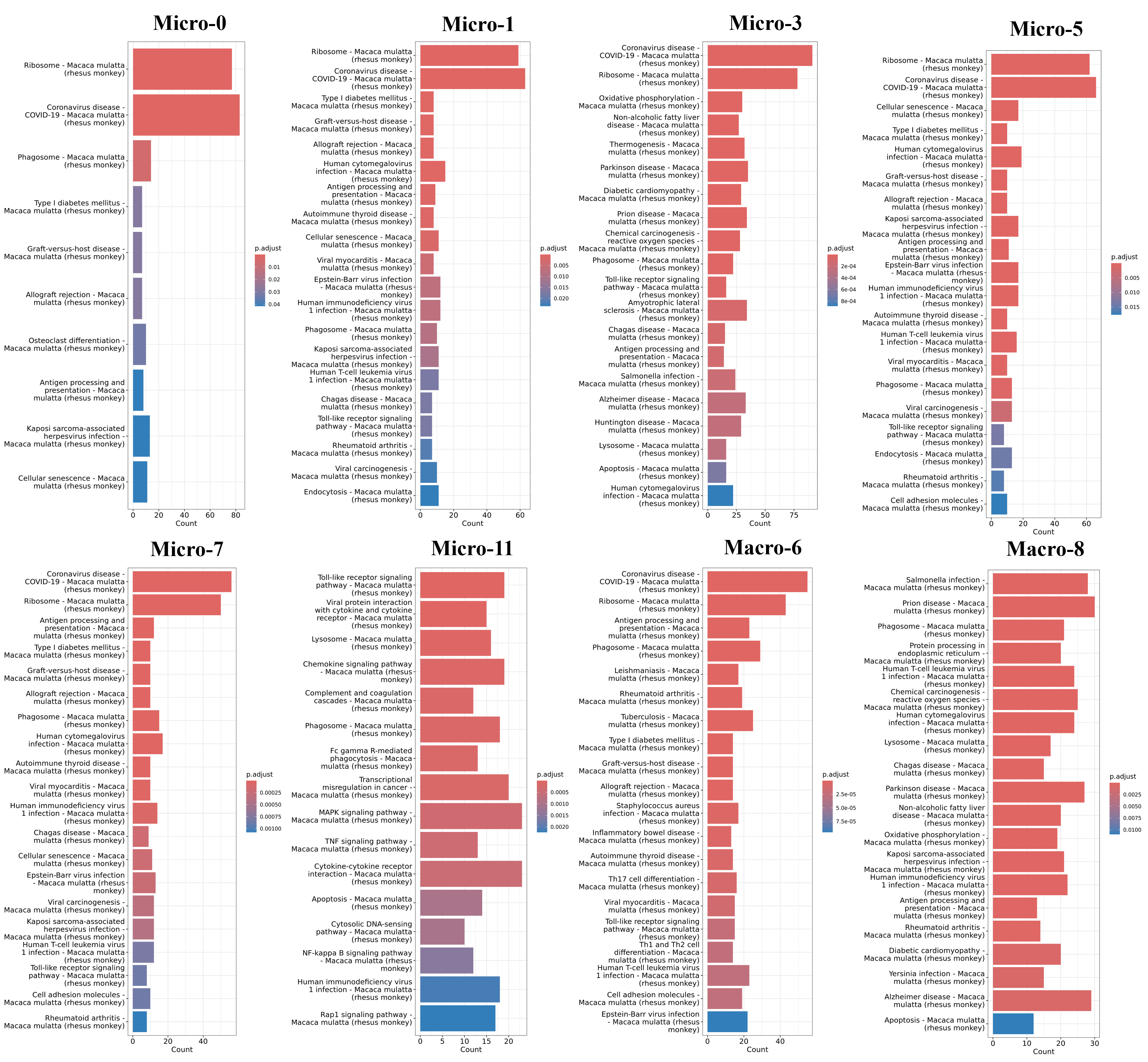

Supplement: S4 Fig — (TIF) [file ppat.1012168.s004.tif]
