# Supplementary material for: Microglia and macrophages alterations in the CNS during acute SIV infection: A single-cell analysis in rhesus macaques
Source: PLoS Pathog. 2024 Sep 16;20(9):e1012168. doi: 10.1371/journal.ppat.1012168 (PMC11426456; doi:10.1371/journal.ppat.1012168)
Supplement: Attachment — Submitted filename: [1] point-to-point responseHF.docx [file ppat.1012168.s011.docx]

Resubmission of *Microglia and macrophages alterations in the CNS during acute SIV infection: a single-cell analysis in rhesus macaques* by Xu *et al.*

Please find our revised manuscript for consideration for publication. We thank the reviewers for their overall positive comments, and we have addressed all concerns as below in the revised manuscript. In response to the issues raised. In addition to edits and text additions/clarifications, we have added a new study for validation of the increase in the Macro_8 cluster, using the cluster marker S100A9 in immunohistochemical studies on tissue sections. As explained below, this has been added to the revised Figure 5 and described in the Results section, as well as added to the Materials and Methods. This work was performed by a student, Shawn Ramachandran, and he has appropriately been added to the author line for this work, for which the other authors agree.

**Please note:** In the pdf file built by the submission site the figures have poor resolution. The proper resolution figures can be accessed by using the link at the top right of each figure on that pdf.

Reviewer 1:

There are several weaknesses. The major weakness is the presentation of a large amount of complex data in multiple figures that are almost illegible in their labeling. There is a great deal of detail in the text about multiple changes in gene expression (DEG), which are difficult to follow without clearer, more legible figures. This generally applies to most/all of the figures. The small number of animals studied (3 experimental, 3 control), which is a common limitation in such primate studies; the investigators recognize this and are appropriately careful in their interpretations.

As below, figures were modified. And yes sample number is a limitation in nonhuman primate studies. The deposition of our data (as well as others’ data) in accessible public databases allows metanalyses to aggregate such data and expand our knowledge.

Other weaknesses are noted. The conclusion that expression of genes in microglia and CAMs reflects SIV infection in those cell types (which is estimated at 0.15%) may be only partially correct. Lymphocytes were not examined in this study, and they may harbor virus at a much higher frequency at this early infection point. Could sub-sets of the microglia and CAMs be affected? This should at least be considered and briefly discussed. Finally, the rationale for choosing a day 12 time point should be discussed specifically with respect to the disease state (day, if possible) being modeled in humans (acute vs. chronic).

These issues are discussed in the responses below.

Other more specific points:

1) In the introduction please update the estimated number of PWH living worldwide—data beyond 2016 should be provided.

The latest epidemiological information for HIV has been updated in the paper, in the Introduction, lines 69-73 in the Track Changes version.

2) Please indicate the estimated time-frame (days or weeks) used in defining acute infection as ‘before the development of antibodies to HIV’. Is the time frame in humans/HIV the same as that in non-human primates/SIV?

In humans, acute HIV infection is typically found 2-4 weeks after exposure. In rhesus monkeys, the acute infection phase for SIVmac251 is typically defined as 1-3 weeks post-infection (pi), given that the viremia peaks at ~14 days pi, and then viremia drops to a steady-state level by 4-6 weeks pi. In general, the time frame for SIV infection (SIVmac251) in rhesus monkeys is parallel with HIV infection in humans. We have included this now in the Introduction lines 82-86, and in the Results lines 155-156 in the Track Changes version.

3) Please also comment or even merely speculate on the relative contribution of lymphocytes vs macrophages in seeding the CNS during this early (12 d) infection. Which predominates? Does the NHP model differ from human infection?

Thank you for pointing this out. This is absolutely correct, on a percentage basis there was a higher proportion of lymphocytes that were infected (~1.4%) than myeloid cells (~0.15%), however there were many more myeloid cells than lymphoid cells. This is now mentioned in the revised manuscript in the Results lines 179-184 in the Track Changes version. In total 68% of the infected cells in the acute stage of infection we studied are myeloid and 32% lymphoid. Because a study on the lymphocytes is out of this paper's scope, we did not delve into this further.

4) Figures should be enlarged. Much of the labeling, axes are illegible.

We apologize. Some figures were enlarged. The axes and legends of feature plot (UAMP embedding) and violin plot were enlarged.

5) The low % of SIV infection in microglia and CAMs (0.15%) at day 12 suggests that perhaps much of the viral load is expressed from lymphocytes. The suggestion that this might reflect the low sensitivity of scRNA-seq and a high false-negative rate is made. Is the inoculating SIV strain macrophage – tropic or strictly lymphocyte-tropic or both?

The SIV strain we injected is SIVmac251 which can infect both CD4+ myeloid cells and lymphoid cells. However, similar to what is observed in HIV infection, CD4+ lymphoid cells were more susceptible to SIVmac251. It is difficult to ascribe for the brain viral load what proportion comes from infected myeloid cells or lymphoid cells, but we do discuss this issue.

6) The assignment of APOE and APOC to neurodegeneration pathways relevant to SIV or HIV pathogenesis is at best speculative. What is true for Alzheimer’s disease or several other neurodegenerative diseases might not be true for this model system. Some references specific for SIV and/or HIV neuropathogenesis risk attributable to APOE and APOC should be provided.

The references have been added to the revised paper and discussed in the Results, lines 342-348 in the Track Changes version.

*Part II – Major Issues: Key Experiments Required for Acceptance*

*Please use this section to detail the key new experiments or modifications of existing experiments that should be absolutely required to validate study conclusions.*

Generally, there should be no more than 3 such required experiments or major modifications for a "Major Revision" recommendation. If more than 3 experiments are necessary to validate the study conclusions, then you are encouraged to recommend "Reject".

Reviewer #1:

1. the observation that SIV infection seems to alter the population size of every microglial and macrophage cluster indicates a global impact of the infection on these cell types. This type of global changes may be of pathogenic significance. Given that microglial in different CNS regions may respond to HIV-1 protein differently (Zheng at el., iScience. 2021 Feb 12;24(3):102186.), I wonder if these changes occur in all brain regions or restricted to regions more relevant to HAND. Immunostaining of some of the cluster markers in selected brain regions would be informative.

Excellent point. Our study on brain myeloid cells was global, from cells purified from approximately half of the brain without separating regions. The new data added to the paper for validation was to do immunostaining for S100A9 as a marker for the Macro-8 cluster. We examined sections from three regions of the brain, frontal lobe, parietal lobe, and temporal lobe, but did not separate into more specific areas such as grey vs white matter, cortex vs basal ganglia, hippocampus, etc. This is presented in the revised Figure 5 (panels C, D, and E in the revised paper, and in the Results lines 531-535 as well as the Discussion lines 693-699 in the Track Changes version.

Our results indeed indicate that there was a significant increase in macrophages expressing this marker in the acutely infected animals, perhaps suggesting that the increase may be global, although the level did seem higher in the section from the temporal lobe. However for rigor we believe that such a finding would need study in much more detail, with better subdivision of brain anatomic structures and more sections examined. We did add a comment on the possibility of regional differences in the revised paper.

2. Micro-3 and macro-8 clusters are with upregulated genes implicated in neurological diseases. This is a potentially important finding. The point would be much stronger if the expression of representative genes in these cells can be confirmed by another approach (e.g., immunostaining or RNAscope).

Agree. One issue was that there were numerous genes enriched in different neuropathological pathways, and many of the common genes enriched in such pathways were related to mitochondria respiration/function. Many of the genes that were upregulated were not necessarily only expressed in one cell cluster, and in some cases also expressed in non-myeloid cells in the brain, so determining which cluster a given cell identified on immunostaining or RNAscope was thus problematic. As in the response to the above point in the revised paper we have verified the increase in cells expression S100A9, a marker for the Macro-8 cluster.

Reviewer #2: The manuscript is outstanding. The n is small but acceptable for the expensive and clarity of the data presented. There is not significant outlawyers for monkey studies. The supplemental tables are well described and organized for other labs to use the large data sets for future studies.

Thank you

Reviewer #3: No new experiments are required, but a major revision of the writing and figure presentation is necessary

We have revised as above

*Part III – Minor Issues: Editorial and Data Presentation Modifications*

*Please use this section for editorial suggestions as well as relatively minor modifications of existing data that would enhance clarity.*

Reviewer #1: it would be informative to include some discussion on the cause of the global changes of all microglial clusters. is it directly caused by the pathogen (SIV)? or pathogen specific?

Since studies of changes in brain myeloid cells with scRNA-seq or other methods that can identify different clusters of microglia cells following infection, we chose not to comment on this.

Reviewer #2: None

Reviewer #3: see above comments
